# Identifying optimal indicators and purposes of population segmentation through engagement of key stakeholders: a qualitative study

**DOI:** 10.1186/s12961-019-0519-x

**Published:** 2020-02-21

**Authors:** Sungwon Yoon, Hendra Goh, Yu Heng Kwan, Julian Thumboo, Lian Leng Low

**Affiliations:** 1 Program in Health Services and Systems Research, Duke-NUS Medical School, Singapore, Singapore; 2 Regional Health System, Singapore Health Services, Singapore, Singapore; 3 Faculty of Science, National University of Singapore, Singapore, Singapore; 4 Department of Rheumatology and Immunology, Singapore General Hospital, Singapore, Singapore; 5 Department of Medicine, Yong Loo Lin School of Medicine, National University of Singapore, Singapore, Singapore; 6 Department of Family Medicine and Continuing Care, Singapore General Hospital, Singapore, Singapore

**Keywords:** Population segmentation, Expert driven, Data driven, Indicator, Purpose

## Abstract

**Background:**

Various population segmentation tools have been developed to inform the design of interventions that improve population health. However, there has been little consensus on the core indicators and purposes of population segmentation. The existing frameworks were further limited by their applicability in different practice settings involving stakeholders at all levels. The aim of this study was to generate a comprehensive set of indicators and purposes of population segmentation based on the experience and perspectives of key stakeholders involved in population health.

**Methods:**

We conducted in-depth semi-structured interviews using purposive sampling with key stakeholders (e.g. government officials, healthcare professionals, social service providers, researchers) involved in population health at three distinct levels (micro, meso, macro) in Singapore. The interviews were audio-recorded and transcribed verbatim. Thematic content analysis was undertaken using NVivo 12.

**Results:**

A total of 25 interviews were conducted. Eight core indicators (demographic characteristics, economic characteristics, behavioural characteristics, disease state, functional status, organisation of care, psychosocial factors and service needs of patients) and 21 sub-indicators were identified. Age and financial status were commonly stated as important indicators that could potentially be used for population segmentation across three levels of participants. Six intended purposes for population segmentation included improving health outcomes, planning for resource allocation, optimising healthcare utilisation, enhancing psychosocial and behavioural outcomes, strengthening preventive efforts and driving policy changes. There was consensus that planning for resource allocation and improving health outcomes were considered two of the most important purposes for population segmentation.

**Conclusions:**

Our findings shed light on the need for a more person-centric population segmentation framework that incorporates upstream and holistic indicators to be able to measure population health outcomes and to plan for appropriate resource allocation. Core elements of the framework may apply to other healthcare settings and systems responsible for improving population health.

**Trial registration:**

The study was approved by the SingHealth Institutional Review Board (CIRB Reference number: 2017/2597).

## Background

Globally, 8% of the population is over 65 years of age, with this figure expected to increase to 20% in 20 years, by which time older persons will outnumber children under the age of 10 (1.41 billion versus 1.35 billion) [1, 2]. Improved quality of life and better healthcare services have contributed to this trend [2, 3]. As a population ages, there is a growing concern of economic burdens associated with chronic diseases, which often entails a tremendous increase in healthcare expenditure [2]. In response, many jurisdictions are developing strategies to reduce the healthcare costs associated with disease burdens and to optimise patient care. One of the strategies is to gain a deeper understanding of the heterogeneous health status and specific healthcare needs of population subgroups, and subsequently assign appropriate healthcare services to each subgroup [4].

Segmentation is a concept typically used to group patients and healthy people into segments with relatively similar needs or characteristics. It is a construct used widely to gauge who might benefit from receiving a certain combination of interventions [5–7]. Research shows that segmentation facilitates the development of an integrated care package of services by implementing tailored care models for each segment [8, 9]. As persons in each segment are relatively similar in terms of healthcare needs, the care package delivered is specific and, at the same time, cost-effective. To this end, segmentation can support policy-makers in measuring outcomes and reducing healthcare costs per capita in each segment [10, 11]. Further, segmentation may contribute to a better understanding of variations within each segment, thereby implementing interventions that are more appropriate [8, 12].

Currently, two major approaches have been developed to segment populations. The first approach is an expert-driven method that segments a population by a priori and expert-defined criteria [13]. Examples of expert-driven approaches include a Senior Segmentation Algorithm, the Bridges to Health model and the North West London model [14–16]. The second approach is a data-driven method that employs post hoc statistical analysis such as clustering analysis or latent class analysis on empirical data to segment a population [13]. Examples of the data-driven method include hierarchical diagnosis models such as the Adjusted Clinical Group System, Classification and Regression Trees and the Clinical Risk Group System [17–19]. Although both methods have been well validated, they do require a comprehensive electronic medical record system [13, 20]. In Singapore, the Ministry of Health proposed a consensus segmentation model, an expert-driven approach to classify patients into five complexity cohorts – healthy, serious acute illness but curable, stable chronic, complex chronic and end of life (Additional file 1).

Despite the apparent value and utility of population segmentation frameworks, effective segmentation is limited by the use of different indicators of segmentation, which may not be grounded in practice settings. In addition, there appears to be a lack of consensus on the purposes of segmentation. One primary reason for the presence of various segmentation frameworks is that population segmentation is performed for different purposes, at times with a limited underlying strategy [21]. Furthermore, existing expert-defined or data-driven frameworks may not necessarily encapsulate the characteristics and needs of a heterogeneous population due to the limited availability of data that can capture the risk factors and holistic care needs of segments [22, 23]. Hence, these gaps have driven the need to develop an actionable population segmentation framework that is well defined in terms of indicators and purposes of segmentation for the whole population in Singapore, with the potential to be generalised beyond the Singapore context.

The overall aim of this study was to generate a comprehensive set of indicators and intended purposes for population health segmentation by engaging key stakeholders involved in population health. Specifically, this study aimed to (1) explore experiences of segmenting population in stakeholders’ areas of work and (2) assess their views of key indicators and purposes to be considered for population segmentation.

## Methods

### Setting

In Singapore, approximately 80% of the population obtain their healthcare services from the public health system. This ranges from inpatient acute conditions to outpatient specialist treatments [24, 25]. In 2018, the government allocated USD $7.5 billion dollars for healthcare expenditure, which were translated into an average of USD $1500 government health expenditure per person [26]. Although government health spending per capita is lower as compared to that of the United States (approximately USD $8000/person), Singapore’s healthcare system continues to rank top in terms of health system efficiency [27]. With improved affordability and quality of healthcare, life expectancy in Singapore is predicted to be 83 years, making it one of the highest in the world. In addition, the percentage of older adults aged 65 years and above in the population is also expected to double to 20% by 2030 [28]. An increased lifespan implies that the number of people with chronic conditions will increase substantially. Naturally, an ageing population would entail a significant increase in healthcare needs. Population segmentation can be utilised to identify cohorts of the population that require more attention and to project long-term care needs.

### Study design and data collection

We conducted semi-structured interviews with a purposive sample of 25 stakeholders involved in population health research and practice. Stakeholders were recruited according to three distinct levels, as follows: (1) macro – policy context in which stakeholders are responsible for developing a population health policy and/or national health infrastructure (e.g. government officials from the Ministry of Health, Agency for Integrated Care, Health Promotion Board and Chief Executive Officers of major restructured hospitals); (2) meso – organisational context in which stakeholders are located at the level of coordination across programmes and implementation of a policy (e.g. population health research analysts, directors of health and social service organisation); (3) micro – context of programme management in which stakeholders are in direct interaction with patients, caregivers and the population (e.g. doctors, nurses and social workers in both community and healthcare settings) [29]. We employed this three-level purposive recruitment to facilitate a holistic and structured analysis. Potential participants were identified via a combination of a snowballing method, where participants suggested key individuals who could provide additional information, and purposively searching individuals in relevant population health areas and/or positions. The sampling frame was broad to ensure that all potential participants were captured. Prior to the commencement of interviews, an interview guide was developed and pre-tested. An email was sent out to potential participants inviting them to participate in the study. Follow-up phone calls were made when necessary. All potential participants approached by the research team consented to take part in one-to-one interviews. The interviews were conducted in the respondent’s workplace by an interviewer trained in qualitative research. The length of each interview ranged from 31 to 104 min.

### Data analysis

All interviews were audio-recorded and transcribed verbatim. Thematic content analysis was conducted to identify a comprehensive set of indicators and purposes of population segmentation in the participant’s own area of work. Two independent coders (SY and HG) carried out open coding and axial coding using NVivo, a qualitative data analysis software. During open coding, transcripts were analysed to develop categories of information. This allowed for subthemes to be derived from the data instead of pre-existing ideas from existing literature or frameworks. During axial coding, common subthemes were grouped into themes. For example, when frailty was discussed extensively by participants, it was selected as one open coding category, positioning it as a central category of the indicators. Then, frailty was subsequently recoded into functional status (axial coding) when similar categories emerged from the data such as patient mobility and activities of daily living in older adults. The iterative process of independent coding and consensus meetings continued until no further new emergent themes were identified. The codes were independently applied to all transcripts and coding discrepancies were resolved by discussion. Employment of a grounded theory approach allowed for conceptual frameworks to be derived from participant inputs. For rigour and transparency, we anchored our methodology according to the Consolidated Criteria for Reporting Qualitative Research (COREQ) checklist [30] (Additional file 2).

## Results

### Characteristics of participants

We interviewed 25 stakeholders. Data saturation was reached after 23 interviews, with no new themes emerging from subsequent interviews. Table 1 shows the characteristics of the 25 participants, including 5 researchers, 9 healthcare professionals, 4 social service providers and 7 government officials; 60% of participants were female and 92% were Chinese. The age range was from 23 to 60 years old, with more than half of the participants (72%) aged 40 years and above. In terms of education, more than two-thirds of the stakeholders (72%) attained postgraduate qualifications. These participants were also balanced in terms of their position and level of involvement in population health – 36%, 32% and 32% were from micro, meso and macro levels, respectively.

**Table 1:** Characteristics of participants (*n* = 25)

Characteristics	N (%)
**Age**	44.36 ± 9.01
< 30	1 (4%)
30–39	6 (24%)
40–49	13 (52%)
50–59	4 (16%)
≥60	1 (4%)
**Ethnicity**	
Chinese	23 (92%)
Malay	1 (4%)
Eurasian	1 (4%)
**Gender**	
Female	15 (60%)
Male	10 (40%)
**Education**	
Bachelor	7 (28%)
Masters	12 (48%)
PhD	6 (24%)
**Profession**	
Government official	7 (28%)
Healthcare professional	9 (36%)
Researcher	5 (20%)
Social service provider	4 (16%)
**Stakeholder grouping**	
Micro	9 (36%)
Meso	8 (32%)
Macro	8 (32%)

### Core indicators of population segmentation

Table 2 showed eight broad domains in relation to the indicators of population health segmentation: demographic characteristics, socioeconomic characteristics, behavioural characteristics, disease state, functional status, organisation of care, psychosocial factors and service needs of patients. The eight domains were further divided into 21 categories. The total number of stakeholders that mentioned certain indicators considered to be important for population segmentation were counted and shaded according to the number of mentions. Shading represented the percentage of stakeholders in each level, reporting the core indicators of population health segmentation. The majority of participants in the macro group agreed that demographic characteristics, such as age and race, should be used to segment the population. Financial status was also considered as an important indicator for population segmentation. While this indicator was mentioned across all levels of participants, it was more strongly expressed by the participants at the macro level. While disease state was commonly mentioned by all participants, certain conditions (e.g. mental health) not typical of indicators in population segmentation frameworks were highlighted. Besides disease state, there was consensus that behavioural characteristics such as awareness of conditions and attitudes towards health-promoting practices were important indicators for population segmentation. Psychosocial factors emerged as one of the important indicators; items include social support from and interaction with family and friends as well as social isolation. Functional status, specifically disability, was reported an important indicator for population segmentation by the majority of participants at the micro level.

**Table 2:**
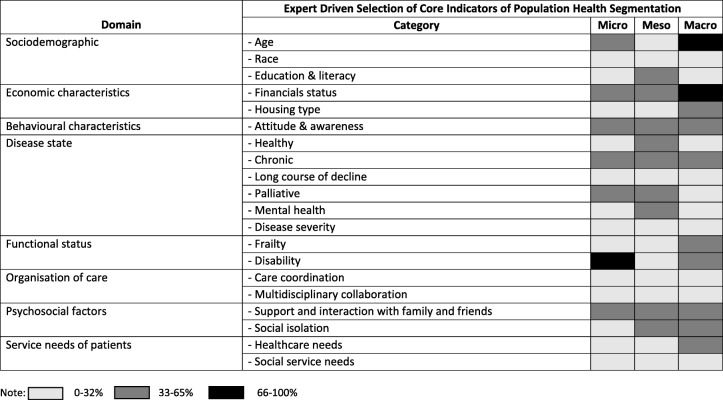
Domains and categories of core indicators of population segmentation

Main themes on the indicators of population segmentation illustrated why certain indicators were selected across different levels of stakeholders. Table 3 showed the stakeholder’s underlying rationale and justification for the selection of particular indicators for segmenting population and the benefits entailed. For example, in the demographic characteristics domain, participants maintained that there was a measurable health disparity across ethnic groups in Singapore, with Malays having lower life expectancy and worse health outcomes as compared to the other two races (i.e. Chinese and Indian). With regards to age as an indicator for population segmentation, participants commonly felt that aging population is a growing concern in Singapore. Inclusion of economic characteristics, in particular housing status, was believed to be important to understand how poverty and inequalities contributed to population health outcomes. More upstream and multiple non-medical determinants of health (social support, service needs, behaviour) were suggested explicitly as many believed that these factors act as a mediator between medical conditions and health outcomes. A minority of participants would want to include the organisation of care as part of the indicators for population segmentation based on their experience of fragmented healthcare delivery.

**Table 3:** Core indicators of population segmentation – themes and illustrative quotes

Domain	Theme	Quotations		
		Micro	Meso	Macro
Demographic characteristics	- Linkage between older age and poorer health outcomes	“*We will focus mainly on the elderly because I think we all know that Singapore is an ageing or rather fast moving to the ageing population. So, we thought that we need to address this from the ground level, from the community*.” #1 Social Service Provider	“*Ageing-related issues are pretty big right? And* [with] *ageing population, people* [are] *living longer, and if we look at* [it] *broader beyond health, how do you keep them engaged? How do you keep them active? What is successful ageing to them?*” #19 Researcher	“*You know, because of the ageing population, the elderly would be a segment of the population that we would want to work on. Which area of the elderly, we will need to work on it a bit more? Because elderly* [the progress] *is a continual right*?” #16 Government Official
	- Health disparity across ethnic groups	“*Actually anybody 40 and above, who can come to the clinic, can be served*.” #1 Social Service Provider	“*We choose fifty-five* [years old] *because it includes sixty-five so it’s a more sensitive measure as opposed to sixty-five where you might exclude some individuals that also need our help*.” #3 Researcher	“*We also do observe particular races like* [the] *Malays as their health outcomes in term of life expectancy are actually much lower compared to other races*.” #20 Government Official
	- Association between low education and increased risk of poor health outcomes	“*I think* [in] *the next 10 years, you’ll probably see more people with more years of education, right? The older people will be so called more educated. It might be easier to for them to be able to access and understand* [healthcare] *information*.” #2 Social Service Provider	“*I would say maybe education level, you know?* [It] *will also protect against certain healthcare.*” #17 Healthcare Professional	“*We’ll have other social factors that affect the population health, for example, housing, education level and literacy*.” #4 Government Official
Economic characteristics	- Level of income affects health outcomes	“*Basically, poverty yeah, no money means not being able to access erm opportunities, or not being able to participate, you know? You can’t go out, you can’t take the transport to go anywhere, you are kind of stuck there*.” #12 Researcher	“*The preliminary findings* [are] *that actually you know patients that are from a lower SES* [socioeconomic status] *group will tend to have more challenges than the rich or rather they are a bit limited to accessing to services. And that has got to do with a lot of social* [factors] *that are* [present] *in their home, that is sort of inhibiting them from access to healthcare*.” #11 Healthcare Professional	“*The lower SES associated with a particular group of race* [is] *basically putting them to poorer health outcomes*.” #20 Government Official
	- Public rental housing and its association with mortality	“*So, for example, whether* [the] *patients* [are] *subsidised, non-subsidised, and sometimes even the residential address could help us to understand the patient’s financial status. If the patient was to come from, for example,* [a] *one room rental property, then that patient* [is] *more likely to be disadvantaged and have more erm social economic issues that will affect their health outcomes*.” #9 Healthcare Professional	“*Our outreach is actually based on three main factors that we always look at. One of the most important factor is HFS* [Home Financial Status].” #10 Researcher	“*We do observe that for err those people who are staying in lower SES conditions, they actually have poorer health outcomes*.” #20 Government Official
Behavioural characteristics	- Health-seeking behaviours as a key determinant of health outcomes	“*Of course, it goes back to the patient’s behaviour and personality. For example, like the ‘meals on wheels’. Some patients need meals on wheels,* [then] *I link you up and you accept it, so there’s no problem. But some will say, ‘Oh I have to pay, you know it’s too expensive, I don’t want it. Oh, I don’t like the food you send to me. I’m going down to buy from the hawker centre.’ You know hawker food is rich in sodium and fats right? Then they’ll have more complications and get readmitted*.” #9 Healthcare Professional	“*They have some health seeking behaviour that influences the health outcome. So this will affect them as to whether they will seek healthcare early or late*.” #6 Healthcare Professional	“*I think different people behave differently. Some people get a deep cut and they think it’s nothing whereas some people might kick up a fuss when they have a small cut. So, it is our personal responses*.” #25 Government Official
Disease state	- Health as a holistic concept	“*We also look at the bio*[logical issues]. W*e look at how the medical condition impacts the patient. Like a dialysis patient. The bio*[logical issue] *is the dialysis, right? So medically, this person needs to go for dialysis. What is this impact of dialysis on the patient? The impact could be it affects their family relationship you know? Because the burden of care is so great, the spouse may decide that you are so burdensome. Every other day I have to bring you to dialysis centre. So, the impact of this dialysis is that the relationship within the family suffers*.” #15 Healthcare Professional	“*Segmenting the population into different categories, from the prefrail, frail, to the complex* [chronic] *care and also the end of life*.” #6 Healthcare Professional	“*People will need help for mental health issues and this is a broad range of issues right? Also, the younger folks that struggle with say depression, schizophrenia, psychosis and all that, so there’s a mental health need group which cuts across all age groups*.” #13 Government Official
	- Tailored healthcare interventions according to disease severity	“*Currently, we’re using the MOH* [Ministry of Health] *tiering, which is Tier 1, Tier 2 and Tier 3. So, Tier 1 are those with stable chronic disease. Then Tier 2 is for those with chronic disease and some geriatric syndrome, some complex nursing and physical needs, functional needs. Tier 3 patients are more complex, and more geriatric syndrome*.” #9 Healthcare Professional	“*Say, for example, illness, right? For example, dementia* [patients]*, they will maybe be of a different group requiring different kind of help*.” #10 Researcher	“*So segment can be in health state like what the British Columbia* [did] *where they have health state: there’s the well, patients who are stable, with chronic diseases, those who are frail, pre-frail or the end of life*.” #16 Government Official
Functional status	- Frailty as a risk factor for poor health outcome	“[To] *prevent the frailty period so that they become strong and can actually age well in the community* [for] *as long as possible*.” #5 Social Service Provider	“*The polyclinic* [government primary care clinic] *also controlled the frails, the elderly who have a lot of problems*.” #19 Researcher	“*We look at the problem of declining function in the elderly, for example, the increasing rate of frailty, the poor control of diseases which lead to more and more complications, the lack of social support leading to readmission*.” #24 Government Official
	- Functional status affects quality of life	“*Well something simple like coping. Can they leave the house? Or can they not leave the house? Because obviously if they cannot leave the house, then that is the sign that something is not quite right*.” #9 Healthcare Professional	“*We measure the functional status as well. Function is not just* [patient] *mobility alone, it can be their ability to function at home, for example, eating, dressing, if they cannot function, it* [will] *affect their quality of life*.” #6 Healthcare Professional	“*I think typically we look at their functional ability. So, seniors who need help with ADL* [activities of daily living] *issues, we help them with grants and subsidy application*.” #13 Government Official
Organisation of care	- Multidisciplinary collaboration improves care coordination	“*Say, for example, if it is an orthopaedic case, then the medical social workers looking after, supporting the orthopaedic team with the knowledge and skills will come in to support them*.” #14 Healthcare Professional	“*Let’s say this patient is seeing a lot of different doctors in a lot of different hospitals, so* [the] *care is a bit fragmented. Usually what would be helpful for these patients is if they have like a regular primary doctor who can liaise with all these different health care professionals, it will help to reduce confusion*.” #3 Researcher	“*We’ll be looking at care coordination. So* [by] *looking at how well is the care being coordinated,* [we can see] *how well the patients are being supported, be it socially or medically outside of the hospital after their discharge*.” #13 Government Official
Psychosocial factors	- Social support promotes general wellbeing	“*Well I think one of the marker is their care. Are they well looked after? If a person is not well looked after, then we would dive in. We will probably dive in to their social and economic issues rather than looking at their medical conditions solely*.” #8 Healthcare Professional	“*It could be* [the] *grandma doesn’t want to take* [the] *medicine, because no one* [is] *supporting grandma. Those problems are the ones that our traditional healthcare is not able to solve. So, we are going to have a lot of healthcare issues that we stare at and it’s a problem that we can’t solve if we just look at it from the healthcare perspective*.” #19 Researcher	“*Poor control of diseases can lead to more and more complications. The lack of social support is one of the key factors leading to readmission*.” #24 Government Official
Service needs of patients	- Identifying patient needs improves delivery of care	“*I would say we go mainly through the needs. Like, for example, the end of life care, what are the needs out there for the end of life care?*” #15 Healthcare Professional	“*By trying to identify patient based on needs is a better reflection of the kind of work we do and recognizing the resources we put into*.” #21 Healthcare Professional	“*To see what kind of services are required for different segments of the population, so segmentation is one way of developing a framework to more coherently decide and identify what are the gaps in terms of care for a particular patient or population segment*.” #16 Government Official

### Purposes for population segmentation

Table 4 shows six broad domains pertaining to the purposes of population segmentation, namely improving health outcomes, planning for resource allocation, optimising healthcare utilisation, enhancing psychosocial and behavioural outcomes, strengthening preventive efforts and driving policy changes. The six domains were further specified into 14 categories. The number of categories mentioned were counted and shaded accordingly. The vast majority of stakeholders across three levels mentioned that the main purpose of segmentation was to allow for better resource management. Specifically, effective use of limited funds and manpower was seen a highly significant purpose for population segmentation. This was followed by optimising healthcare utilisation, more specifically, reducing the frequency and length of hospitalisation. Strengthening preventive efforts emerged as an important domain. This domain was more salient in meso and macro groups. Expectedly, stakeholders from the macro group commonly felt that population segmentation was an important driver for policy changes.

**Table 4:**
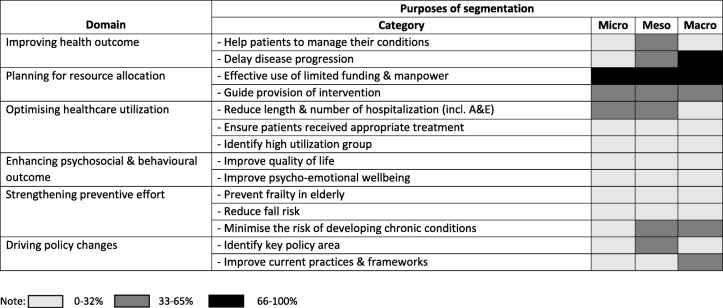
Domains and categories of purposes for population segmentation

The main themes and illustrative quotes on the purposes of segmentation are presented in Table 5. Participants across three levels agreed that the primary reason for segmenting the population was to measure the impact of the existing healthcare interventions. Thus, they would want to see improved medical outcomes such as delaying disease progression and improving healthy aging. Repeated hospital admissions, longer waiting times and unnecessary medical treatments appeared to be key concerns for most of the participants. As such, it was generally believed that, in terms of service delivery, the direct output of population segmentation was to identify high healthcare user segments, thereby introducing appropriate interventions to address their care needs. As one participant in micro group pointed out, effective identification of ‘frequent flyers’ through segmentation would enable care providers to target patients’ needs, which could then be translated into efficient use of healthcare resources. Another theme running through the data was that a well segmented population would provide insight into better health policy planning, especially in terms of healthcare financing. As one government official reported, segmentation could help health authorities to identify the low-income, high-risk subgroups, which can inform the design of policies that provides affordable healthcare for these segments. By and large, the intended purposes for segmentation varied by the type of stakeholders. Government officials would want to allocate limited resources more effectively by segmenting the population, whereas frontline clinicians and social service providers would hope to tailor care management services for the identified segments.

**Table 5:** Purposes for population segmentation – themes and illustrative quotes

Domain	Theme	Quotations		
		Micro	Meso	Macro
Improving health outcomes	- Delay progression of chronic conditions	“*Basically, we want to promote and advocate for positive ageing experience at the end of the day*.” #2 Social Service Provider	“*So, for residents who have three or more chronic diseases, who require assistance to manage the chronic diseases, and if they are able to walk, they will come down to the nursing post to seek consultation from my nurses and for those who can’t walk, then my nurses will go up to their house*.” #6 Healthcare Professional	“*Helps us to focus on a few key things on how the health system is able support the person in doing some of these things well so that they don’t transit into a worse state*.” #13 Government Official
	- Care of patients with multi-morbidity	“*One of the outcomes will be: Did they get their service can improve their health? You know, for example, if someone needs dialysis and needs to be referred to the NKF* [National Kidney Foundation]*, we make sure that it is done*.” #14 Healthcare Professional	“*We look at the usual length of stay, number of discharges,* [the] *number of dead. Not everybody dies here you know? Some people do go home*.” #23 Healthcare Professional	“*Because if someone is in the, let’s say, a pre-frail stage, can we do something to delay their movement into their worse stage*.” #25 Government Official
	- Healthy aging through self-care			
Planning for resource allocation	- Identify high resource consumption groups	“*So, they try to think about* [how] *to reduce wastage in terms of resource allocation*.” #12 Researcher	“*It tells us that* [with] *whatever limited resources and time you have, you are addressing patient’s needs. Patient’s needs are prioritised so at least you know you are helping that patient*.” #11 Healthcare Professional	“*So, the question is where should we direct the resources to get the most return for the money we put in? Basically, having all these indicators right from the start and seeing which are performing well, which are not performing well,* [it] *could help us to know exactly where to target*.” #20 Government Official
	- Reduce inappropriate medical treatments	“*For the patients, they don’t get unnecessary treatment and for the institution, you don’t actually waste money and resources. Then, for countrywide right, you actually saved on financial expenditure and healthcare planning.*” #22 Healthcare Professional	“*Ideally, you have identified a population segment that is fairly substantial like a big chunk of your interest population and they have interventions available for them that is fairly effective. Then wouldn’t this be a place that you want to put money in and give them the improvements in health outcome that is possible through the service you have?*” #3 Researcher	“*So* [for] *resources allocation, the whole notion about population segmentation is actually to be able to use of our resources wisely*.” #17 Government Official
Optimising healthcare utilisation	- Assess impact of healthcare interventions on hospital readmission	“*Of course, for the vulnerable elderly, we will be looking at the hospital admissions*.” #5 Social Service Provider	“*I mean the hospital admission rates is important for prognosis.* [If] *somebody keeps being admitted regularly in the hospital, it tells you something. Either they’re not coping at home so it’s a huge social problem, or the disease is getting worse. So, it does tell you a lot about the patient*.” #23 Healthcare Professional	“*Because the purpose of segmentation is the risk of readmission and then you intervene and monitor the rate of readmission. Has the rate of readmission gone up? Gone down? Or never change at all? So, if you have the model, you have the intervention and the readmission drops, I won’t say it is a true cause and effect yet there’s a correlation*.” #24 Government Official
	- Identify and manage high utilisation group	“*These are indicators that they can be easily measured. Like the rate of readmission and the length of stay. How often do people come back to emergency department? How often do they default treatment?*” #15 Healthcare Professional	“*We look at utilisation status after segmenting, to see which group consumed the most healthcare resources*.” #23 Healthcare Professional	“*It allows us to identify segments of needs so existing programmes like H to H* [Hospital to Home] *has very high resource utilisation as a segment.*” #16 Government Official
Enhancing psychosocial and behavioural outcomes	- Improve patient’s quality of life	“*Of course, there is* [the] *emotional wellbeing. We track their attendances* [to our activities] *so that we know* [how] *connected they are to* [the] *community. This is the outcome that we look for under the well elderly*.” #5 Social Service Provider	“*For example, in end of life group, the focus is to make sure that we optimise their quality of life at this point in time, rather than to focus on treating or prolonging their life*.” #7 Researcher	“*So, we will be looking at outcomes such as reduced disability and increased quality of life till the end of life*.” #13 Government Official
	- Promote psychosocial wellbeing			
Strengthening preventive effort	- Reduce fall risk to improve health outcomes	“*We look at their frailty score because our wellness programme targets on reversing or preventing frailty. As you know a fall is a major risk factor for death, especially for elderly living alone*.” #5 Social Service Provider	“*The second group is people that have different types of chronic diseases. Then the goal is actually to optimise their chronic disease management so that we are able to delay or prevent complications*.” #7 Researcher	“*We are only delaying the frailty because we want him to stay on in his home rather than readmitted to the hospital, when most of the time it will take a very long time for him to recover*.” #13 Government Official
	- Prevent disease complications			
Driving policy changes	- Facilitate government funding on specific key areas	“*Well, for example, the senior mobility fund. Because senior mobility fund is for 65 and above and they* [must be] *means tested in order to qualify for subsidies for care aid and mobility aid. If this patient exceeds the means testing by $1, technically this means that he is not qualified for the subsidy. But do you think that the $1 is really that significant? So, we hope that whatever we are doing now could influence policy changes.*” #9 Healthcare Professional	“*You can actually have the data and if the evidence supports it, it can facilitate policy planning in healthcare financing. For example, patients with high BPS* [biopsychosocial] *score will have better capitated funding or better funding for their GP visit*.” #23 Healthcare Professional	“*We probably would have to identify key areas to work on, focus area. Again, how this is done then leads into a larger policy framework*.” #16 Government Official
	- Target previously neglected healthcare needs		“*If you can get all these* [healthcare utilisation data] *and then study the data, this could be a way to inform the ministry* [on] *how to finance primary care properly*.” #21 Healthcare Professional	“*If we found* [out] *that lower income families are having poorer outcomes because of affordability, then we need to craft out more policies to help in terms of affordability*.” #20 Government Official

## Discussion

This study aimed to develop an actionable population segmentation framework that incorporated the views and experiences of key stakeholders involved in population health. To our knowledge, this study was the first to generate a comprehensive set of indicators and purposes important for population segmentation by engaging key stakeholders across three different levels of involvement in population health.

### Core indicators of segmentation

Our findings revealed eight broad domains as potential core indicators in population segmentation. As expected, one of the most commonly mentioned indicators was ‘disease state’. It was believed that, by grouping people with similar health conditions together, the disease state allowed policy-makers to develop interventions specifically targeting segment groups and promoting positive health outcomes [31, 32]. Indeed, in the current segmentation framework in Singapore, health conditions are used to segment a population as a sole indicator. This method tends to draw criticism for being overly disease centred and selective; one example would be the exclusion of ‘mental health’ in the framework reported by many of our participants. In general, there was consensus amongst our participants on the need for person centricity, a shift away from the disease-centred approach, to account for social and environmental features. Nonetheless, psychosocial and behavioural indicators are not routinely collected locally for population health management. In contrast, the Northwest London segmentation model (Additional file 1), a segmentation framework developed by the Whole System Integrated Care in United Kingdom, incorporated mental conditions into the framework [33]. Hence, the framework enabled the development and evaluation of mental health interventions, as evidenced by the Penn Resiliency Program, a group intervention programme delivered to children below 15 years old to improve resiliency skills and optimistic thinking [34]. The Northwest London segmentation framework was also deemed holistic since it allowed for more homogeneous groupings of the population into smaller clusters [35]. However, if mental health was established as a single stratum on its own, significant uncertainty can arise with respect to the right boundaries for segmentation [36]. For example, it may be difficult to categorise a patient with both chronic kidney disease and depression if both conditions were to be a stand-alone category. Therefore, more work and considerations would be required to identify optimal ways to segment disease states.

Our findings showed that ‘age’ under the socioeconomic domain was one of the important indicators of population segmentation. This finding was supported by the Northwest London segmentation model, which incorporated age as one of the segmentation criteria [33]. By contrast, age was not accounted for in Singapore’s segmentation framework. It is widely known that aging leads to an impairment to physical function and increase in mortality [37]. It has also been well established that the incidence of chronic disease rises sharply with age and that the majority of patients with chronic conditions are over the age of 65 years [38]. Therefore, based on the risk of developing chronic diseases, the population can be segmented into different age groups. For the younger population (< 21 years old), health education and screening programmes could be introduced as an early intervention tool. Beitz et al. pointed out that there is a positive association between health education and disease prevention [39]. Therefore, if younger generations become more health literate, the progression of common chronic diseases can be prevented or delayed [40]. In a similar fashion, appropriate preventative health programmes can reach out to persons aged between 40 years and 60 years. This is a stage where many adults start experiencing major changes in their lives [41, 42]. To enable this segment of population to effectively manage their conditions, interventions such as chronic disease screening and counselling could prove beneficial [40, 43]. By segmenting the population into a more granular extent, it would not only enhance prognosis but also reduce healthcare expenditure [44]. However, a study by Wood et al. noted that, unlike common understanding of the utility of age for population segmentation, chronic conditions rather than age itself were found to be a better indicator of healthcare expenditure [32]. Hence, caution is warranted when including age as an indicator of segmentation.

‘Financial status’ was also shown to be a major indicator of population segmentation. Being diagnosed with a chronic disease can have a significant financial strain on the family, depleting the household’s resources [45]. As healthcare costs continue to increase relative to household income, it will compete with basic household expenditure and cause financial hardship for families [46]. This situation might be worsened if a patient is unemployed or a family member has to reduce employment obligations as a result of caregiving responsibilities [47]. Taking into consideration the implications of financial instability when segmenting the population would allow policy efforts to focus more on alleviating the economic burden of illness on households [48]. In Singapore, a healthcare assistance scheme, the Community Health Assist Scheme, is available for all Singapore citizens with chronic conditions [49]. The scheme is tiered according to monthly household income or the Annual Value of a home as assessed by the Inland Revenue Authority to enable people, particularly from lower- and middle-income households, to receive subsidies for medical and dental care [49]. Hence, segmenting population according to financial status not only supports the lower- to middle-income class families to cope with their medical expenses, but the segmentation also allows the Ministry of Health to ensure the affordability of healthcare while improving health outcomes [50].

### Purposes for population segmentation

Our findings indicated that one of the most commonly stated purposes for population segmentation was ‘delaying the progression of diseases’ and ‘improving self-management of health conditions’. Hence, health outcomes across the care pathway as well as those off the care pathway (i.e. people that need the care but are not receiving it) need to be considered in order to identify intervention priorities. In this sense, segmentation can inform the design of evidence-based interventions and evaluation by examining longitudinal changes in the health profiles of each population segment [15, 51]. For example, the National Health Service in the United Kingdom developed a chronic care model to improve patient activation towards self-care and disease management, to ultimately delay progression of chronic diseases [52]. Hence, collective improvement in disease prognosis indicated that treatments and care were effective for the targeted groups. Almost all stakeholders in the macro group identified this to be an important purpose of segmentation. It may be that, as this group comprised primarily policy-makers and government officials, they wished to be reassured that healthcare interventions should benefit population health [51].

Another important purpose of segmentation was the ‘effective use of limited funding and workforce’. With population segmentation, the number of people and corresponding healthcare expenditures for each segment can be quantified. This would allow identifying the gap in the existing services and ensuring the right number of healthcare professionals to be trained [53]. For example, a study by Anne et al. demonstrated an effective nurse-led preventive intervention. The success of the intervention was attributed to the segmentation of older patients according to their health conditions in response to the growing prevalence of frailty and functional decline [54]. As the total number of patients to be served was identified prior to the intervention, segmentation allowed the estimation of the total number of nursing posts needed to optimally serve this pool of patients. This finding is well mirrored in the Bridges to Health model, one of the established models for population segmentation (Additional file 1) [55]. The model was developed with the purpose of providing effective healthcare services to meet the varying needs of different population subgroups while reducing healthcare costs [56].

Overall, our findings supported current literature stating that the expert-defined approach to population segmentation requires more than just a health condition to enhance its applicability. For example, Wood et al. found that the expert-driven binning models, such as Bridges to Health, are unlikely to achieve high levels of discrimination between cohorts even though it has easily interpretable segments and could be useful for benchmarking [32]. Likewise, the data-driven approach to population segmentation entailed a considerable risk of bias as many data-driven tools typically rely on administrative record data, which are often incomprehensive [57]. Therefore, it is important that good segmentation approaches take into consideration the factors associated with applicability, discrimination and practicality [32]. The key to a successful segmentation model would also lie in valuing insights from stakeholders at all levels and providing a sound rationale capable of implementation [58].

### Strengths and limitations

This study has several strengths. We identified shortcomings in the existing frameworks used for segmenting population in Singapore and in other settings. Our study demonstrated that a set of indicators that had been used in the electronic health record system might not be sufficient, given the diverse population that the stakeholders served in various contexts. Stakeholders also provided relevant insights on core indicators to develop a robust population segmentation framework. The lack of routine collection of such core indicators and the limited applicability of the framework in different practice settings can pose challenges to the successful implementation of evidence-based practice in population health. Hence, this study contributes to the potential development of an actionable segmentation framework that is both relevant in the context of Singapore’s population and that of advanced economies. In addition, stakeholders interviewed were from the three levels of population health, ranging from those interacting with patients directly to people involved in healthcare institutions and then health policy-makers. This enabled us to cross-validate responses and facilitated consensus views of what indicators and purposes were important at different levels of population health.

Notwithstanding the strengths, our findings should be considered in light of a few limitations. Despite efforts to limit methodological bias, findings from this were derived from qualitative research, which was by nature prone to a degree of potential subjectivity. The way the participants were categorised into three levels of analysis revealed an important insight into population segmentation. Nevertheless, it was possible that other researchers would have made different decisions around defining categories, for example, in terms of professional background or area of work.

## Conclusion

In conclusion, this study has identified a comprehensive set of indicators and purposes of population segmentation through engagement of key stakeholders for the population in Singapore, with the potential of being generalised beyond the Singapore context. In addition, we have discussed issues and shortcomings surrounding the existing segmentation frameworks that are being used. Findings from this study shed light on the need for a more person-centric framework that incorporates upstream and holistic indicators to be able to measure population health outcomes and to plan for appropriate resource allocation.

## Supplementary information

**Additional file 1: Figure S1.** Ministry of Health segmentation framework. **Figure S2.** Northwest London segmentation model. Description for Northwest London segmentation model. **Figure S3.** Bridges to Health model. Scenario for Bridges to Health model.

**Additional file 2.** Consolidated criteria for reporting qualitative research (COREQ): 32-item checklist.

## Data Availability

All data generated or analysed during this study are included in this published article and its supplementary information files.

## References

[1] Berrío Valencia MI (2012). Envejecimiento de la población: un reto para la salud pública. Revista Colombiana de Anestesiología.

[2] Low LL, Kwan YH, Liu N, Jing X, ECT L, Thumboo J (2017). Evaluation of a practical expert defined approach to patient population segmentation: a case study in Singapore. BMC Health Serv Res.

[3] Christensen K, Doblhammer G, Rau R, Vaupel JW (2009). Ageing populations: the challenges ahead. Lancet.

[4] Phelps C, Madhavan G, Rappuoli R, Levin S, Shortliffe E, Colwell R (2016). Strategic planning in population health and public health practice: a call to action for higher education. Milbank Q.

[5] Keleher H (2011). Planning for population health in Australia’s health reforms. Aust N Z J Public Health.

[6] Schoen C, Osborn R, How SK, Doty MM, Peugh J (2009). In chronic condition: experiences of patients with complex health care needs, in eight countries, 2008. Health Aff.

[7] Yan S, Kwan YH, Tan CS, Thumboo J, Low LL (2018). A systematic review of the clinical application of data-driven population segmentation analysis. BMC Med Res Methodol.

[8] Vuik SI, Mayer EK, Darzi A (2016). Patient segmentation analysis offers significant benefits for integrated care and support. Health Aff.

[9] Tynan AC, Drayton J (1987). Market segmentation. J Mark Manag.

[10] Yan S, Seng BJJ, Kwan YH, Tan CS, Quah JHM, Thumboo J, Low LL (2019). Identifying heterogeneous health profiles of primary care utilizers and their differential healthcare utilization and mortality – a retrospective cohort study. BMC Fam Pract.

[11] Yan S, Kwan YH, Thumboo J, Low LL (2019). Characteristics and health care utilization of different segments of a multiethnic Asian population in Singapore. JAMA Netw Open.

[12] Chong JL, Matchar DB (2017). Benefits of population segmentation analysis for developing health policy to promote patient-centred care. Ann Acad Med Singap.

[13] Low LL, Yan S, Kwan YH, Tan CS, Thumboo J (2018). Assessing the validity of a data driven segmentation approach: a 4 year longitudinal study of healthcare utilization and mortality. PLoS One.

[14] Lynn J, Straube BM, Bell KM, Jencks SF, Kambic RT (2007). Using population segmentation to provide better health care for all: the “Bridges to Health” model. Milbank Q.

[15] Vuik SI, Mayer E, Darzi A (2016). A quantitative evidence base for population health: applying utilization-based cluster analysis to segment a patient population. Popul Health Metrics.

[16] Zhou YY, Wong W, Li H (2014). Improving care for older adults: a model to segment the senior population. Perm J.

[17] Hughes JS, Averill RF, Eisenhandler J, Goldfield NI, Muldoon J, Neff JM, Gay JC (2004). Clinical Risk Groups (CRGs): a classification system for risk-adjusted capitation-based payment and health care management. Med Care.

[18] Reid RML, Roos NP, Bogdanovich B, Black C (1999). Measuring morbidity in populations: performance of the John Hopkins Adjusted Clinical Group (ACG) case-mix adjustment system in Manitoba.

[19] Sabine Ingrid Vuik EM, Darzi A (2016). Understanding population health needs: how data-driven population segmentation can support the planning of integrated care. Int J Integr Care.

[20] Chong JL, Low LL, Chan DYL, Shen Y, Thin TN, Ong MEH, Matchar DB (2019). Can we understand population healthcare needs using electronic medical records?. Singapore Med J.

[21] Lega F, Mengoni A (2012). Profiling the different needs and expectations of patients for population-based medicine: a case study using segmentation analysis. BMC Health Serv Res.

[22] Solomatine D, See LM, Abrahart RJ. Data-driven modelling: concepts, approaches and experiences. Data Driven Approach. 2008;2:17–30.

[23] Ramzan M, Jaffar MA, Shahid AA (2011). Value based intelligent requirement prioritization: expert driven fuzzy logic based prioritization technique. Int J Innov Comput Inform Control.

[24] How CH, Fock KM (2014). Healthcare in Singapore: the present and future. Singap Med J.

[25] Khoo HS, Lim YW, Vrijhoef HJ (2014). Primary healthcare system and practice characteristics in Singapore. Asia Pac Fam Med.

[26] Tan A (2018). Singapore budget 2018: spending needs to grow in healthcare, infrastructure, security and education. The Business Times.

[27] Ting WP (2018). S’pore’s healthcare system best in value and satisfaction, but falls behind in providing access: study. Today.

[28] Ong B (2019). The changing healthcare landscape in Singapore – setting the context. NUS Newsletter.

[29] Sheri Pruitt SA (2002). Innovative care for chronic conditions.

[30] Tong A, Craig J, Sainsbury P (2007). Consolidated criteria for reporting qualitative research (COREQ): a 32-item checklist for interviews and focus groups. Int J Qual Health Care.

[31] Stratton E, Lampit A, Choi I, Calvo RA, Harvey SB, Glozier N (2017). Effectiveness of eHealth interventions for reducing mental health conditions in employees: a systematic review and meta-analysis. PLoS One.

[32] Wood RM, Murch BJ, Betteridge RC (2019). A comparison of population segmentation methods. Oper Res Health Care.

[33] Bailey C, Paice E (2016). North West London Whole Systems Integrated Care: a case study. Int J Integr Care.

[34] Kobau R, Seligman MEP, Peterson C, Diener E, Zack MM, Chapman D, Thompson W (2011). Mental health promotion in public health: perspectives and strategies from positive psychology. Am J Public Health.

[35] Bailey C (2013). Case study: North West London – profiling the population according to need.

[36] Holmes AM, Deb P (2004). Performance assessment in community mental health care and at-risk populations. Health Care Financ Rev.

[37] Probst-Hensch NM (2010). Chronic age-related diseases share risk factors: do they share pathophysiological mechanisms and why does that matter?. Swiss Med Wkly.

[38] Lavrovsky Y, Chatterjee B, Clark RA, Roy AK (2000). Role of redox-regulated transcription factors in inflammation, aging and age-related diseases. Exp Gerontol.

[39] Beitz JM (1998). Education for health promotion and disease prevention: convince them, don't confuse them. Ostomy Wound Manage.

[40] Lee S, Huang H, Zelen M (2004). Early detection of disease and scheduling of screening examinations. Stat Methods Med Res.

[41] Oles PK (1999). Towards a psychological model of midlife crisis. Psychol Rep.

[42] Wong LP, Awang H, Jani R (2012). Midlife crisis perceptions, experiences, help-seeking, and needs among multi-ethnic malaysian women. Women Health.

[43] Ellman JP (1992). A treatment approach for patients in midlife. Can J Psychiatr.

[44] Schweitzer SO (1974). Cost effectiveness of early detection of disease. Health Serv Res.

[45] Cunningham P, Carrier E (2014). Trends in the financial burden of medical care for nonelderly adults with diabetes, 2001 to 2009. Am J Manag Care.

[46] Paul C, Boyes A, Hall A, Bisquera A, Miller A, O'Brien L (2016). The impact of cancer diagnosis and treatment on employment, income, treatment decisions and financial assistance and their relationship to socioeconomic and disease factors. Support Care Cancer.

[47] Arozullah AM, Calhoun EA, Wolf M, Finley DK, Fitzner KA, Heckinger EA, Gorby NS, Schumock GT, Bennett CL (2004). The financial burden of cancer: estimates from a study of insured women with breast cancer. J Support Oncol.

[48] Patel MR, Piette JD, Resnicow K, Kowalski-Dobson T, Heisler M (2016). Social determinants of health, cost-related nonadherence, and cost-reducing behaviors among adults with diabetes: findings from the national health interview survey. Med Care.

[49] Wee LE, Cher WQ, Sin D, Li ZC, Koh GC-H (2016). Primary care characteristics and their association with health screening in a low-socioeconomic status public rental-flat population in Singapore- a mixed methods study. BMC Fam Pract.

[50] Deborah OML, Chiu MYL, Cao K (2018). Geographical accessibility of community health assist system general practitioners for the elderly population in Singapore: a case study on the elderly living in housing development board flats. Int J Environ Res Public Health.

[51] Scrivens E, Cunningham D, Charlton J, Holland WW (1985). Measuring the impact of health interventions: a review of available instruments. Eff Health Care.

[52] Wagner EH (2004). Chronic disease care. BMJ.

[53] Garzonis K, Mann E, Wyrzykowska A, Kanellakis P (2015). Improving patient outcomes: effectively training healthcare staff in psychological practice skills: a mixed systematic literature review. Eur J Psychol.

[54] Marcus-Varwijk AE, Peters LL, Visscher TLS, Smits CHM, Ranchor AV, Slaets JPJ (2020). Impact of a nurse-led health promotion intervention in an aging population: results from a quasi-experimental study on the “Community health consultation offices for seniors”. J Aging Health.

[55] Lynn J, Straube BM, Bell KM, Jencks SF, Kambic RT (2007). Using population segmentation to provide better health care for all: the “Bridges to health” model. Milbank Q.

[56] Schoenbaum SC, Gauthier AK, Koren MJ (2007). Commentary on the “Bridges to health” model. Milbank Q.

[57] Chong JL, Lim KK, Matchar DB (2019). Population segmentation based on healthcare needs: a systematic review. Syst Rev.

[58] O'Malley AS, Rich EC, Sarwar R, Schultz E, Warren WC, Shah T, Abrams MK (2019). How accountable care organizations use population segmentation to care for high-need, high-cost patients. Issue Brief.

