# Supplementary material for: Identifying optimal indicators and purposes of population segmentation through engagement of key stakeholders: a qualitative study
Source: Health Res Policy Syst. 2020 Feb 21;18:26. doi: 10.1186/s12961-019-0519-x (PMC7035731; doi:10.1186/s12961-019-0519-x)
Supplement: Supplementary file 1 — Additional file 1: Figure S1. Ministry of Health segmentation framework. Figure S2. Northwest London segmentation model. Description for Northwest London segmentation model. Figure S3. Bridges to Health model. Scenario for Bridges to Health model. [file 12961_2019_519_MOESM1_ESM.pptx]

## Slide 1
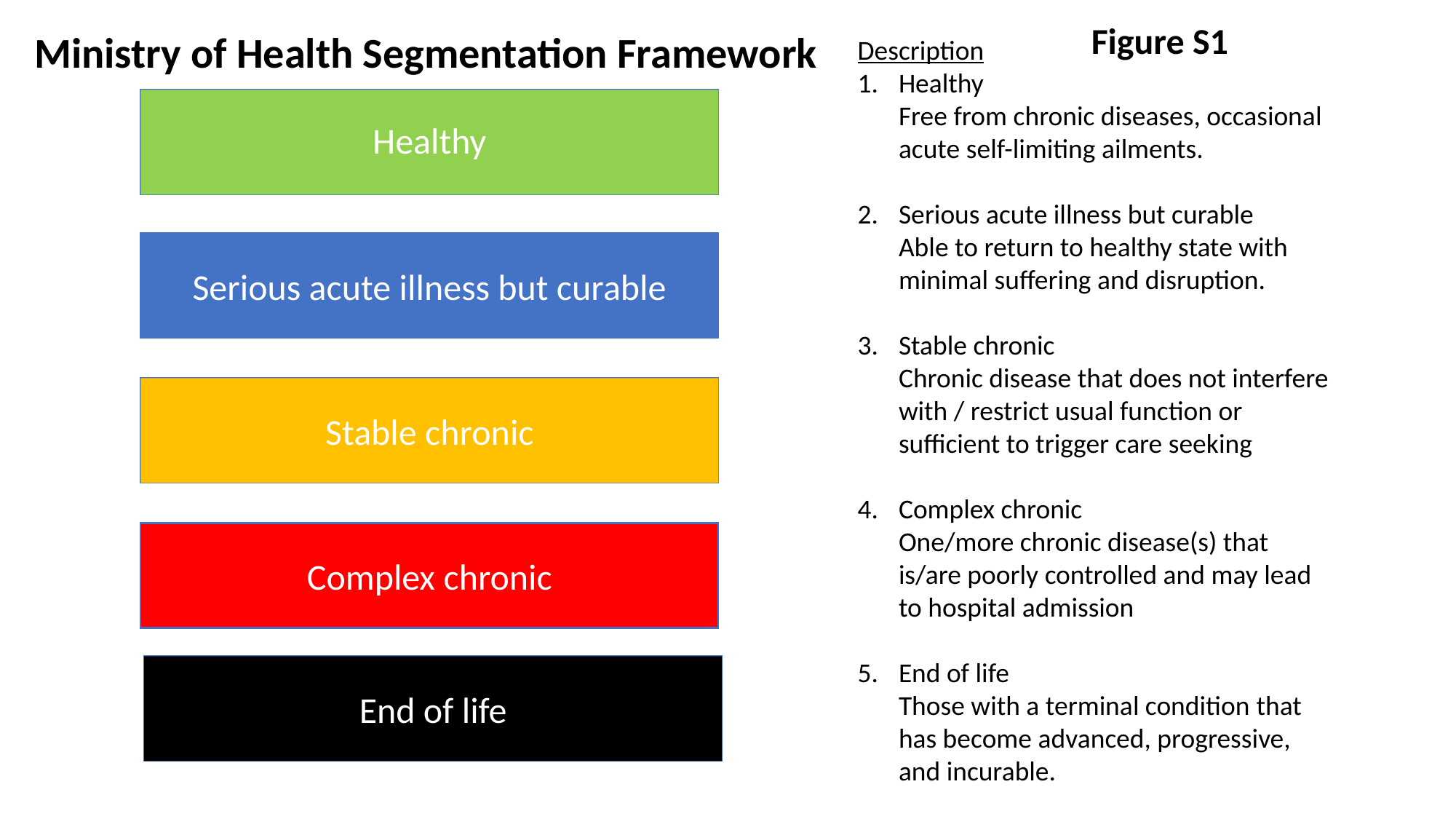

Figure S1
Ministry of Health Segmentation Framework
Description
Healthy
Free from chronic diseases, occasional acute self-limiting ailments.
Serious acute illness but curable
Able to return to healthy state with minimal suffering and disruption.
Stable chronic
Chronic disease that does not interfere with / restrict usual function or sufficient to trigger care seeking
Complex chronic
One/more chronic disease(s) that is/are poorly controlled and may lead to hospital admission
End of life
Those with a terminal condition that has become advanced, progressive, and incurable.
Healthy
Serious acute illness but curable
Stable chronic
Complex chronic
End of life

## Slide 2
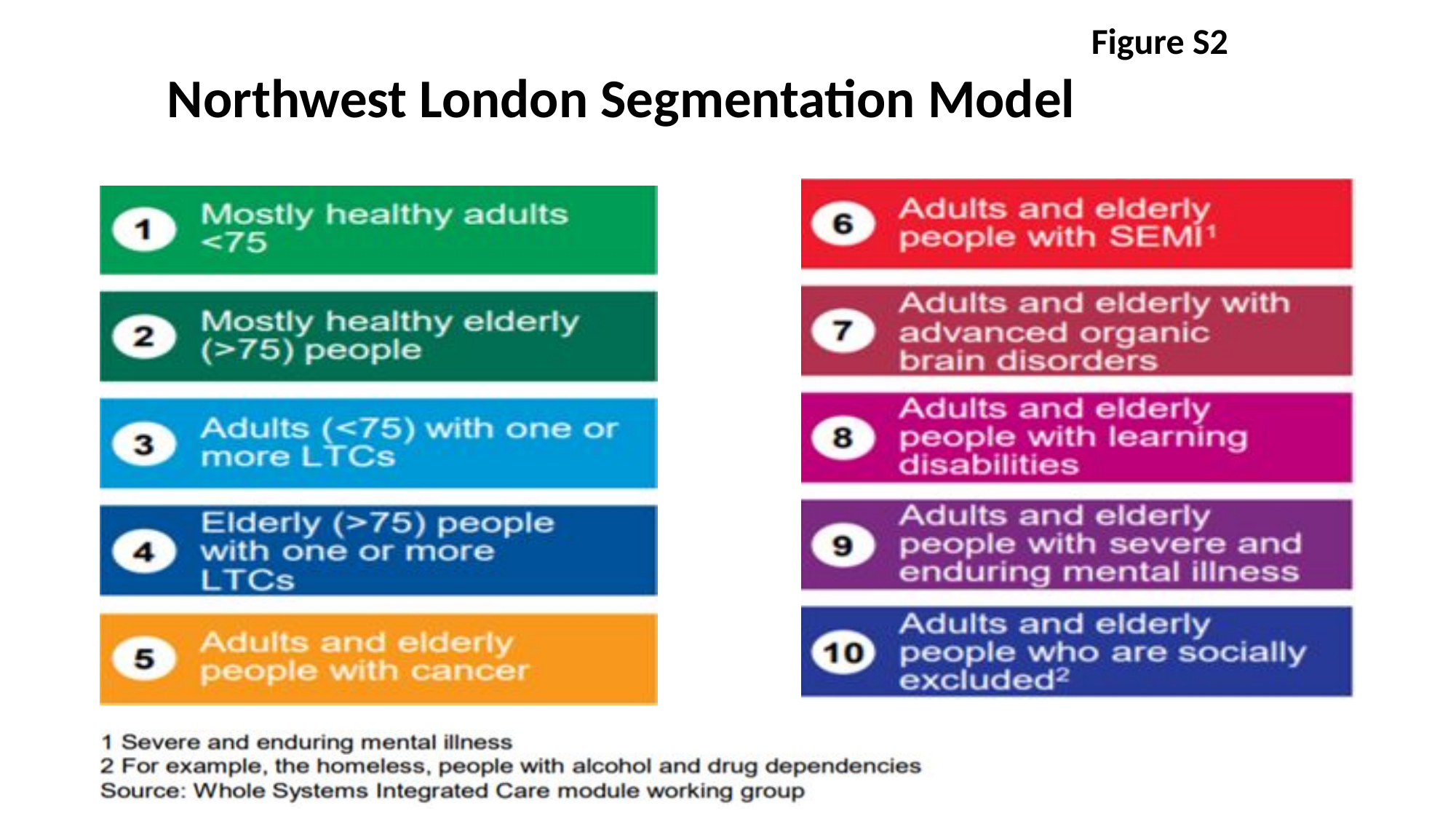

Figure S2
# Northwest London Segmentation Model

## Slide 3
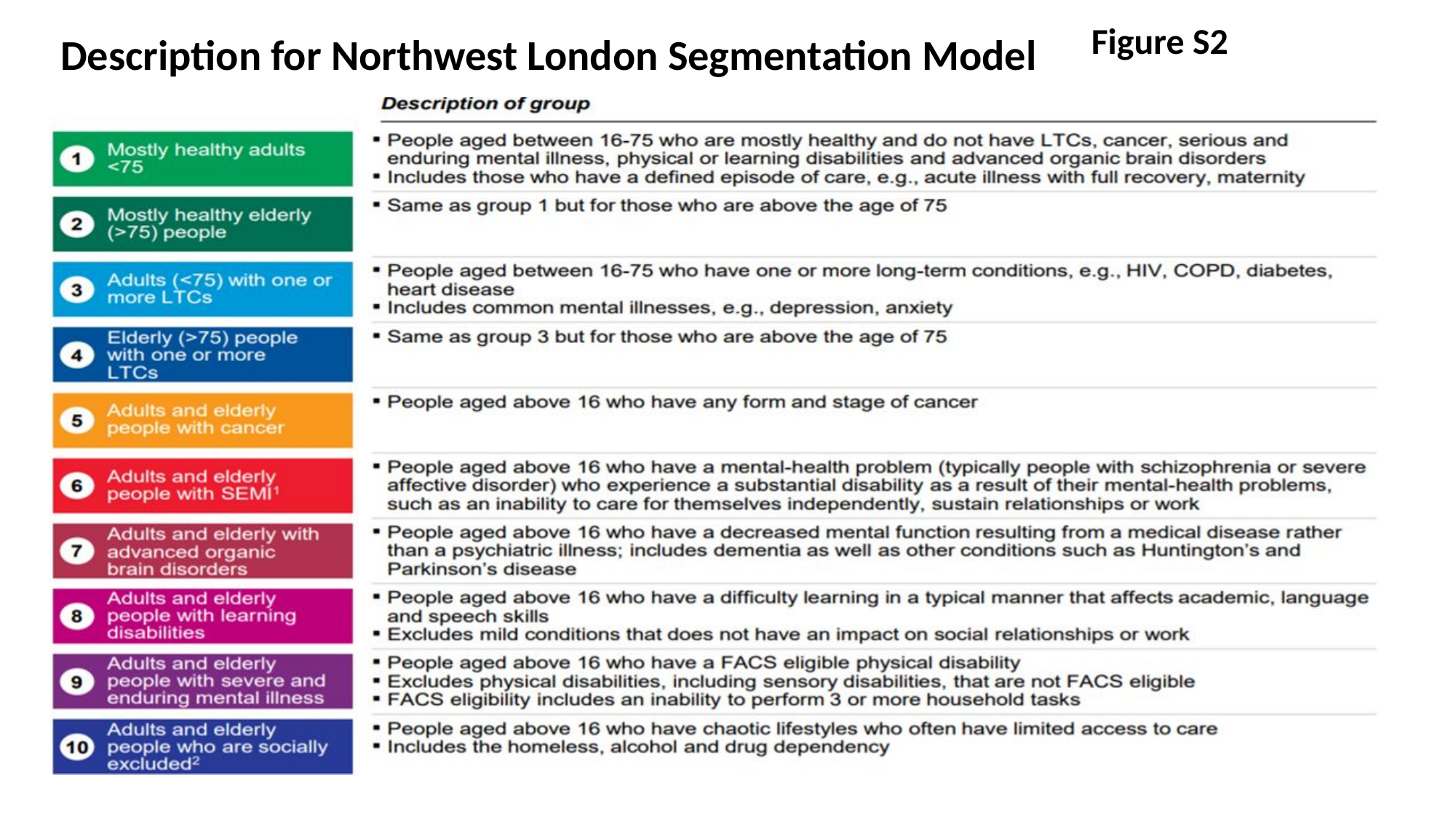

Figure S2
Description for Northwest London Segmentation Model
#

## Slide 4
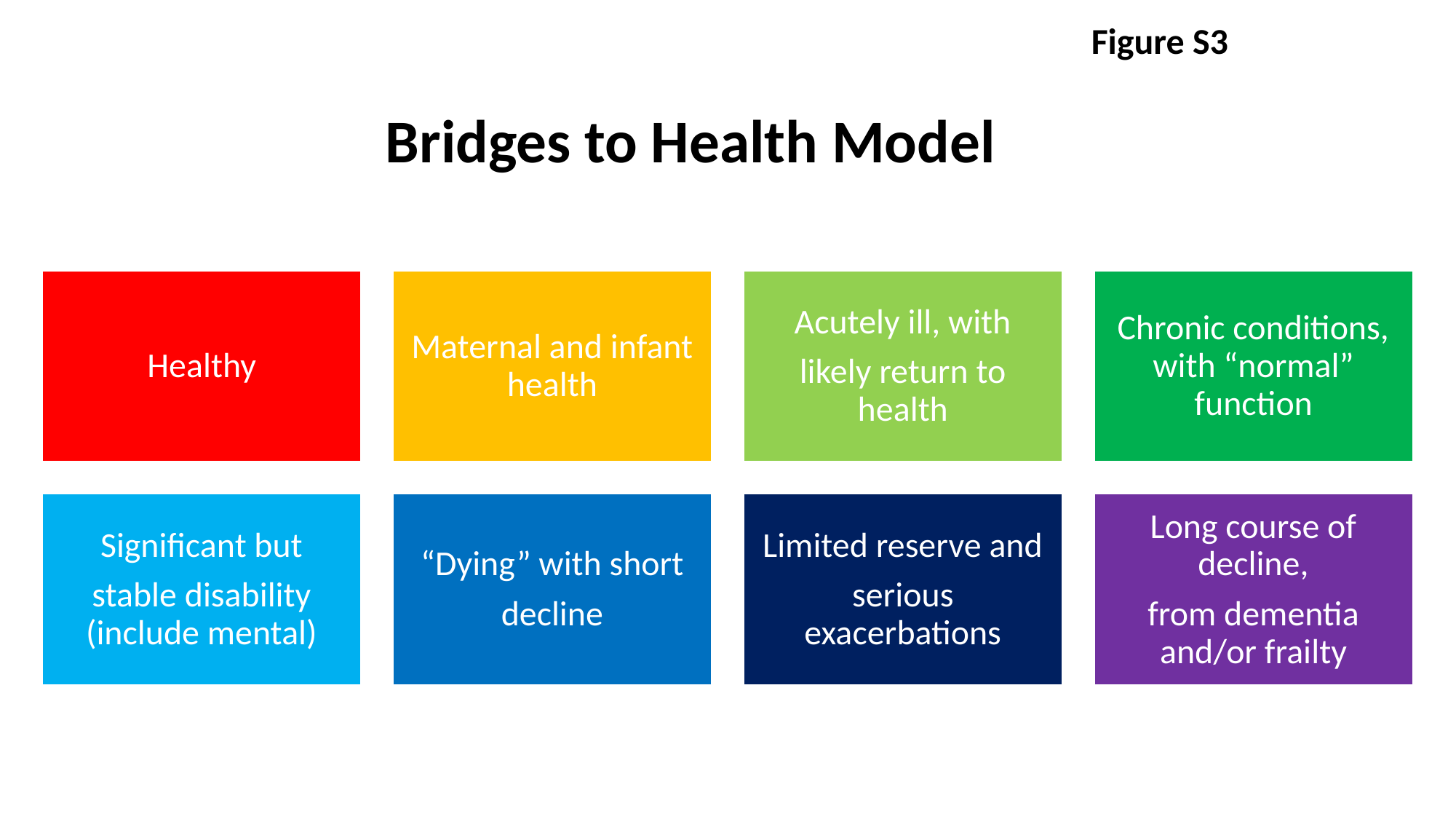

Figure S3
# Bridges to Health Model

## Slide 5
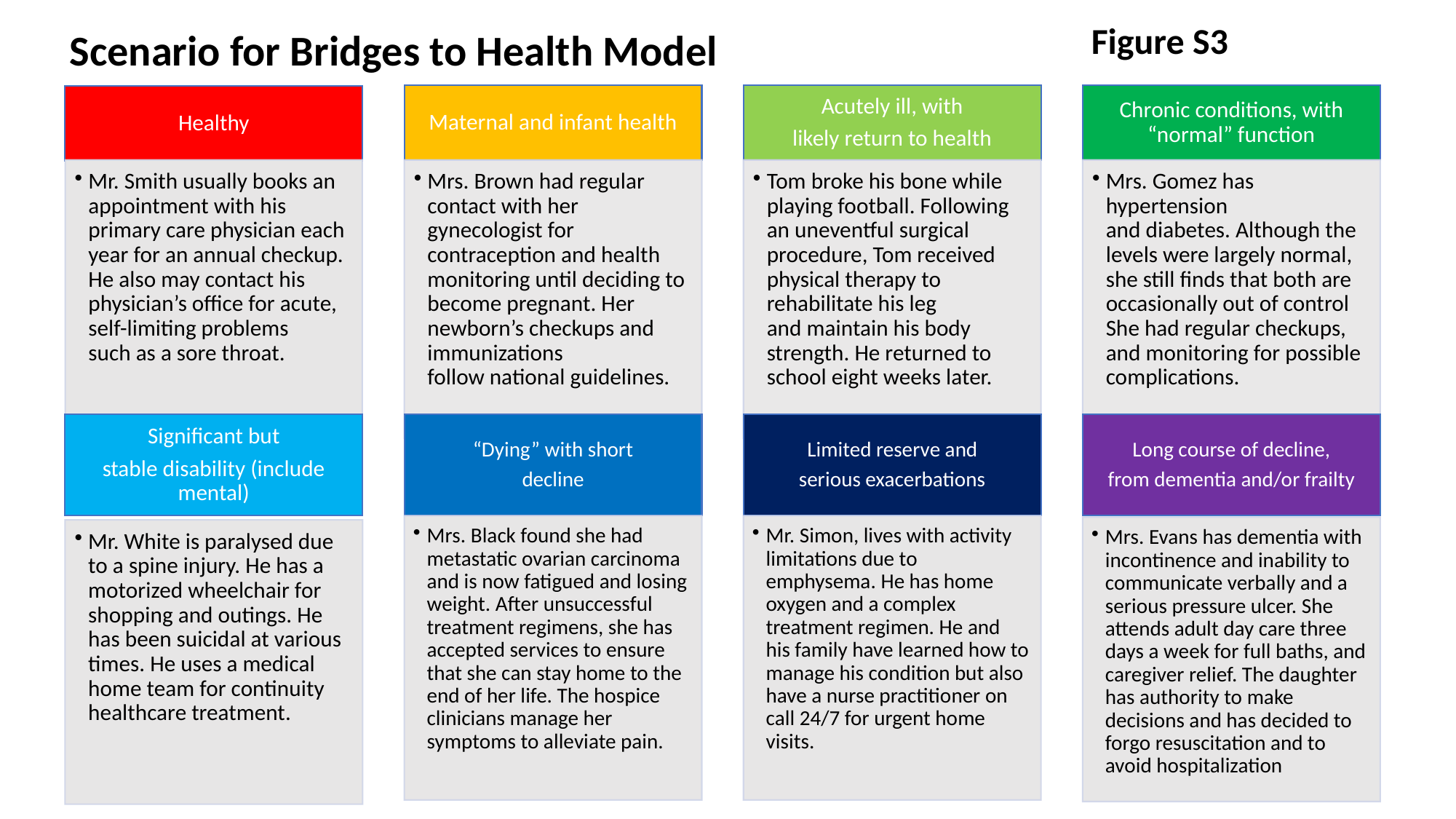

Figure S3
Scenario for Bridges to Health Model
